# A novel 12-membered ring non-antibiotic macrolide EM982 attenuates cytokine production by inhibiting IKKβ and IκBα phosphorylation

**DOI:** 10.1016/j.jbc.2024.107384

**Published:** 2024-05-16

**Authors:** Rui Saito, Hisanori Domon, Takumi Hiyoshi, Satoru Hirayama, Tomoki Maekawa, Shoji Takenaka, Yuichiro Noiri, Akari Ikeda, Tomoyasu Hirose, Toshiaki Sunazuka, Yutaka Terao

**Affiliations:** 1 Division of Microbiology and Infectious Diseases, Niigata University Graduate School of Medical and Dental Sciences, Niigata, Japan; 2 Division of Cariology, Operative Dentistry and Endodontics, Department of Oral Health Science, Niigata University Graduate School of Medical and Dental Sciences, Niigata, Japan; 3 Center for Advanced Oral Science, Niigata University Graduate School of Medical and Dental Sciences, Niigata, Japan; 4 Division of Periodontology, Niigata University Graduate School of Medical and Dental Sciences, Niigata, Japan; 5 Kitasato Institute for Life Sciences, Kitasato University Graduate School of Infection Control Sciences, Kitasato University, Tokyo, Japan

**Keywords:** macrolide derivatives, antibiotics, immunosuppression, Toll-like receptor 4 (TLR4), cell signaling, drug development

## Abstract

Antimicrobial resistance poses a serious threat to human health worldwide and its incidence continues to increase owing to the overuse of antibiotics and other factors. Macrolide antibiotics such as erythromycin (EM) have immunomodulatory effects in addition to their antibacterial activity. Long-term, low-dose administration of macrolides has shown clinical benefits in treating non-infectious inflammatory respiratory diseases. However, this practice may also increase the emergence of drug-resistant bacteria. In this study, we synthesized a series of EM derivatives, and screened them for two criteria: (i) lack of antibacterial activity and (ii) ability to suppress tumor necrosis factor-α (TNF-α) production in THP-1 cells stimulated with lipopolysaccharide. Among the 37 synthesized derivatives, we identified a novel 12-membered ring macrolide EM982 that lacked antibacterial activity against *Staphylococcus aureus* and suppressed the production of TNF-α and other cytokines. The effects of EM982 on Toll-like receptor 4 (TLR4) signaling were analyzed using a reporter assay and Western blotting. The reporter assay showed that EM982 suppressed the activation of transcription factors, NF-κB and/or activator protein 1 (AP-1), in HEK293 cells expressing human TLR4. Western blotting showed that EM982 inhibited the phosphorylation of both IκB kinase (IKK) β and IκBα, which function upstream of NF-κB, whereas it did not affect the phosphorylation of p38 mitogen-activated protein kinase, extracellular signal-regulated kinase, and c-Jun N-terminal kinase, which act upstream of AP-1. These results suggest that EM982 suppresses cytokine production by inhibiting phosphorylation of IKKβ and IκBα, resulting in the inactivation of NF-κB.

The World Health Organization (WHO) has declared antimicrobial resistance (AMR) as a global public health threat. An estimated 1.27 million deaths were directly attributable to bacterial AMR in 2019 (1). Additionally, diseases associated with AMR are expected to cause 10 million deaths each year by 2050 unless extensive and concerted action is taken to reduce the emergence and spread of AMR (2). In this regard, the WHO has developed a global action plan for AMR, including the strategic objective of optimizing antibiotic use.

Macrolides such as erythromycin (EM), clarithromycin, and azithromycin are antibiotics that exhibit broad-spectrum activity against several bacterial species by inhibiting protein synthesis. They are commonly used to treat various bacterial infections (3). WHO classifies macrolides as “watch” antibiotics in the AWaRe classification (4), indicating a need for limited use due to a high risk of resistance. Despite this recommendation, national or regional surveillance of antibiotic consumption indicates that macrolides still represent a significant portion of total antibiotic consumption in many countries (5, 6, 7). This trend persists because macrolides, in addition to their antibacterial activity, have immunomodulatory properties. Consequently, they have found clinical application in “non-infectious” chronic inflammatory diseases such as diffuse pan-bronchiolitis, chronic obstructive pulmonary disease, asthma, and bronchiectasis, supported by substantial evidence (8, 9). However, the therapeutic use of macrolides due to their immunomodulatory effects increases the risk of AMR. Thus, despite their immense therapeutic potential, AMR poses a challenge to effectively utilizing the immunomodulatory properties of macrolides.

We have previously developed various novel EM derivatives (10, 11). In this study, we screened derivatives that lacked antibacterial activity but possessed immunomodulatory properties and analyzed their mechanisms of action to identify non-antibacterial derivatives and avoid AMR expansion risk.

## Results

**Figure 1:**
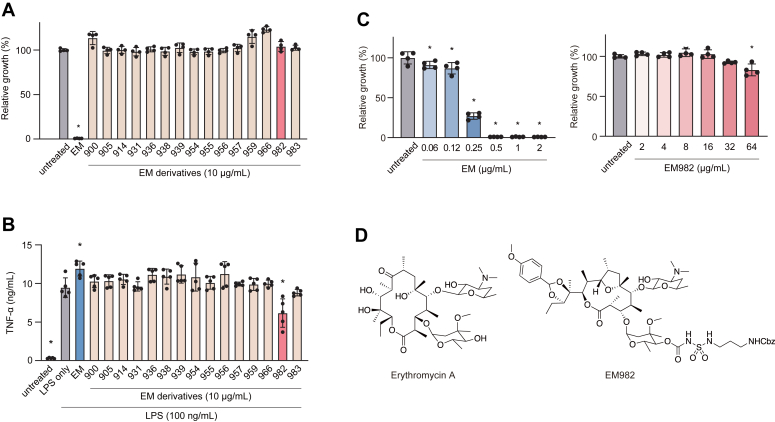
**Identification of a novel macrolide with immunomodulatory effects and very low antibacterial activity.***A*, antibacterial activity of erythromycin (EM) derivatives was analyzed. Macrolide-sensitive *Staphylococcus aureus* strain NILS6 was cultured in the presence or absence of 10 μg/ml EM or EM derivatives for 24 h. Bacterial proliferation was quantified by spectrometry at 620 nm. Ctrl; control. *B*, The immunomodulatory effect of non-antibacterial EM derivatives was analyzed. THP-1-derived macrophages were incubated in the presence or absence of 10 μg/ml EM or EM derivatives for 2 h prior to stimulation by 100 ng/ml LPS for 8 h. After incubation, TNF-α level in the culture supernatant was measured by ELISA. *C*, minimum inhibitory concentrations of EM and EM982 against *S. aureus* NILS6 were determined. *A–C*, the data represent the mean ± SD of samples per group and were evaluated by one-way ANOVA with Dunnett's multiple comparisons test. The asterisks indicate significant differences as compared with (*A* and *C*) untreated group or (*B*) LPS-only group (∗*p* < 0.05). *D*, structures of erythromycin A and EM982. Erythromycin A is a 14-membered ring antibacterial macrolide. EM982 is a novel 12-membered ring non-antibacterial macrolide synthesized from erythromycin A.

To screen for non-antibacterial EM derivatives, we tested their ability to inhibit the growth of EM-sensitive *Staphylococcus aureus* strain NILS6. The EM900 series and most of the other series tested in this study did not inhibit the growth of *S. aureus* (Figs. 1*A* and S1). This indicates the absence of or low antibacterial activity of these derivatives. To identify EM derivatives with immunomodulatory properties, we then investigated the effect of derivatives on lipopolysaccharide (LPS)-induced tumor necrosis factor (TNF)-α production in THP-1 cells. As shown in Figure 1*B*, only EM982 suppressed the production of TNF-α compared to the LPS-only group. Next, we compared the antibacterial activities of EM982 and EM. The minimum inhibitory concentration (MIC) of EM against *S. aureus* NILS6 was 0.5 μg/ml, whereas EM982 exhibited only slight growth inhibition even at 64 μg/ml (Fig. 1*C*). MICs of EM and EM982 were compared against other bacterial species and strains. Similar to the results of NILS6, the MICs for these strains differed substantially between EM and EM982 (Table S1). These results suggest that EM982 has minimal antibacterial activity, but exhibits superior immunosuppressive activity.

The structures of EM and EM982 are shown in Figure 1*D*. Erythromycin was chemically modified to develop EM900, a 12-membered ring non-antibacterial macrolide. EM900 and its series have been reported to exhibit potent anti-inflammatory and immunomodulatory effects *in vitro* and *in vivo* (10, 11). The novel derivative EM982 was synthesized to serve as a chemical probe for EM900.

**Figure 2:**
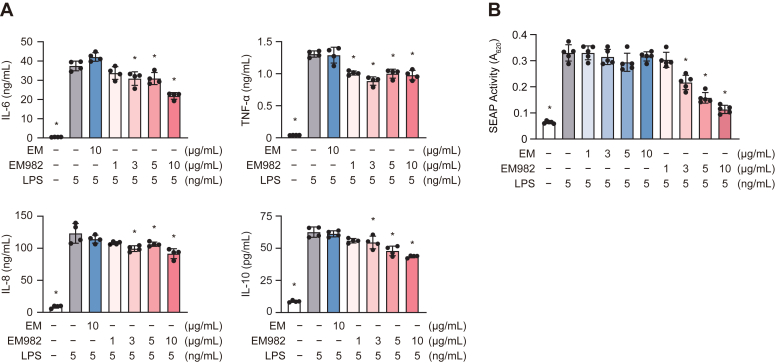
**EM982 inhibited activation of NF-κB or AP-1, resulting in suppression of cytokine production from THP-1 cells.***A*, THP-1 macrophages were incubated in the presence or absence of 10 μg/ml EM or 1 to 10 μg/ml EM982 for 2 h prior to stimulation by 5 ng/ml LPS for 12 h. Cytokine levels in the culture supernatant were measured by ELISA. *B*, HEK-Blue hTLR4 cells were cultured with 1 to 10 μg/ml EM or EM982 for 2 h prior to stimulation by 5 ng/ml LPS for 15 h. The activity of SEAP was quantified by spectrometry at 620 nm. *A* and *B*, the data represent the mean ± SD of samples per group and were evaluated by one-way ANOVA with Dunnett's multiple comparisons test. The *asterisks* indicate significant differences as compared with the LPS-only group (∗*p* < 0.05).

We further analyzed the effects of EM982 on the production of other cytokines, including interleukin (IL)-6, IL-8, and IL-10, in LPS-stimulated THP-1 macrophages. Compared with the LPS-only group, EM982 (≥3 μg/ml) significantly decreased the concentration of all cytokines in the culture supernatant, whereas EM did not exhibit a significant effect on cytokine production (Fig. 2*A*). To determine the molecular mechanisms by which EM982 decreases the production of these cytokines, we focused on NF-κB and activator protein 1 (AP-1), which are transcription factors that play a central role in cytokine transcription. Secretory embryonic alkaline phosphatase (SEAP) reporter assays were used to analyze the effect of EM982 on NF-κB and AP-1 activity in response to LPS stimulation in HEK-Blue cells expressing human Toll-like receptor 4 (HEK-Blue hTLR4 cells; refer to Experimental procedures). Compared to the LPS-only group, EM982 (≥3 μg/ml) significantly downregulated the SEAP activity, whereas EM did not (Fig. 2*B*). These results suggest that EM982 suppresses the TLR4/NF-κB and/or TLR4/AP-1 pathways.

To analyze which molecules in these pathways were affected by EM982, we performed real-time PCR on 84 signaling molecules using TaqMan Array Plates. The geNorm algorithm applied to the PCR data determined that HPRT-1 was the most stable of the 12 reference genes (Fig. S2). The relative quantity of mRNA was normalized to that of *HPRT1* mRNA, and the fold changes in mRNA levels in EM982-treated THP-1 cells were calculated relative to those in the LPS-only group. Thus, the relative quantities of mRNA were normalized to those of *HPRT1* mRNA, and fold changes of mRNA level in EM982-treated THP-1 cells were calculated relative to that of the LPS-only group. The fold changes of all molecules were not more than two or less than half, suggesting that EM982 had little effect on the transcription of any molecule (Table 1).

**Table 1:** Among the TLR signaling molecules, none showed high transcriptional variation in EM982-treated THP-1 cells

Gene	Fold change
NF-κB signaling	
*BTK*	1.04
*CHUK*	0.82
*IKBKB*	0.91
*IKBKE*	1.00
*IKBKG*	0.88
*IRAK1*	1.42
*IRAK2*	0.84
*IRAK4*	1.00
*MYD88*	1.04
*NFKB1*	0.86
*NFKB2*	0.91
*NFKBIA*	0.79
*NFKBIB*	0.82
*NFKBIE*	0.90
*REL*	0.87
*RELA*	1.08
*RELB*	0.98
*RIPK1*	0.95
*RIPK2*	1.40
*RIPK3*	0.99
*TANK*	1.07
*TBK1*	1.01
*TRAF6*	ND^a^
TLRs, coreceptors and adaptors	
*CD14*	0.99
*LBP*	ND^a^
*LY96*	0.99
*TICAM1*	1.22
*TICAM2*	0.91
*TIRAP*	0.96
*TLR1*	1.20
*TLR10*	ND^a^
*TLR2*	1.03
*TLR3*	1.12
*TLR4*	1.17
*TLR5*	ND^a^
*TLR6*	ND^a^
*TLR7*	1.20
*TLR8*	ND^a^
*TLR9*	ND^a^
Negative regulator	
*ECSIT*	0.95
*IL1R1*	ND^a^
*IRAK3*	0.98
*RNF216*	0.93
*SIGIRR*	0.98
*TOLLIP*	1.03
MAPK signaling	
*MAP2K3*	0.93
*MAP2K6*	1.03
*MAP2K7*	0.88
*MAP3K3*	1.05
*MAP3K7*	0.91
*MAP3K7IP1*	1.04
*MAP3K7IP2*	0.96
*MAPK10*	0.90
*MAPK11*	1.37
*MAPK12*	1.19
*MAPK13*	1.05
*MAPK14*	0.72
*MAPK8*	0.88
*MAPK9*	1.05
Other transcription factors	
*ATF2*	0.97
*ATF4*	0.91
*CREB1*	0.99
*CREB3*	0.96
*CREB3L4*	1.13
*IRF3*	1.01
*IRF7*	1.02
*IRF8*	0.89
*JUN*	0.94
PI3K signaling	
*PIK3C2A*	1.00
*PIK3C2B*	ND^a^
*PIK3C3*	ND^a^
*PIK3CA*	0.90
*PIK3CB*	0.97
*PIK3CD*	1.41
*PIK3R1*	1.01
*PIK3R2*	1.02
*PIK3R3*	0.82
*PIK3R4*	1.02
*PIK3R5*	0.95
*RAC1*	1.01

a ND, not detected. Genes with no detection or very low expression (Ct value ≥ 35) were considered as ND.

**Figure 3:**
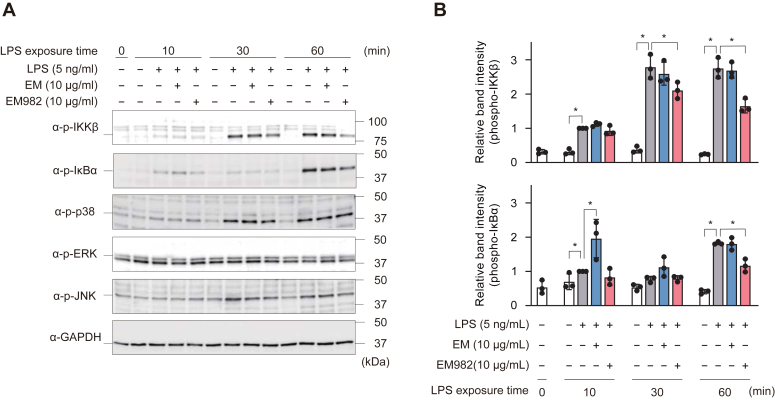
**EM982 exerts immunomodulatory effects by downregulating the NF-κB signaling pathway.***A*, THP-1-derived macrophages were incubated in the presence or absence of 10 μg/ml EM or 10 μg/ml EM982 for 2 h prior to stimulation by 5 ng/ml LPS for 0 to 60 min. The expression of phospho-IKKβ (p-IKKβ), p-IκBα, p-p38, p-ERK, and p-JNK were detected by Western blotting. Representative images were shown. *B*, band intensities of p-IKKβ and p-IκBα were quantified and normalized against that of GAPDH. The data represent means ± SD of triplicate experiments and were evaluated by one-way ANOVA with Dunnett's multiple comparisons tests. The *asterisks* indicate significant differences as compared with the LPS-only group (∗*p* < 0.05).

Next, western blotting was performed to analyze the levels of TLR4 signaling molecules. EM982 inhibited the phosphorylation of IκB kinase (IKK) β and IκBα, which act upstream of NF-κB, at 30 to 60 and 60 min, respectively, compared to the LPS-only group (Fig. 3). However, EM982 did not affect the phosphorylation of p38 mitogen-activated protein kinase (p38), extracellular signal-regulated kinase (ERK), and c-Jun N-terminal kinase (JNK), which mainly act upstream of AP-1, suggesting that EM982 inhibits only the NF-κB pathway. These data suggest that EM982 affected the activity of IKKβ or its upstream molecules, thereby inhibiting NF-κB activation through decreased IκBα phosphorylation and degradation.

Western blotting showed that EM982 suppressed the NF-κB pathway but not the AP-1 pathway. To confirm these findings, we performed an additional SEAP reporter assay using THP-1-Blue cells to evaluate only NF-κB activity. Fig. S3 shows that EM982 suppressed SEAP activity in phorbol myristate acetate (PMA)-untreated THP-1-Blue cells, suggesting that the immunomodulatory effect of EM982 is mediated by suppressing the NF-κB pathway. We then analyzed cytokine production in PMA-untreated THP-1 cells. These cells required 200-fold higher concentrations of LPS to induce cytokine production equivalent to THP-1 macrophages. EM982 decreased IL-6 and IL-8 production (Fig. S4), consistent with that shown in Figure 2 but did not decrease TNF-α production. EM decreased IL-6 and IL-8 production only in PMA-untreated THP-1 cells (Fig. S4). IL-10 levels in the supernatant of PMA-untreated THP-1 cells were below the detection limit (data not shown).

## Discussion

Macrolide antibiotics exert an immunomodulatory effect on the host’s immune function, including the regulation, especially downregulation, of excessive inflammation without impairing the normal immune response for biological defense, unlike the action of steroids and other drugs (12). Since the first reports on the immunomodulatory effects of macrolides, various macrolides have been reported to be clinically effective in improving symptoms and quality of life, as well as reducing the risk of acute exacerbations in patients with chronic inflammatory respiratory diseases, such as diffuse panbronchiolitis, chronic obstructive pulmonary disease, asthma, and bronchiectasis (13, 14, 15, 16, 17, 18). Based on accumulated evidence, the British Thoracic Society guidelines clearly state that long-term low-dose administration of macrolides is a therapeutic option for these diseases (19). Additionally, macrolide therapy can be effective against bacterial infections such as bacterial pneumonia and periodontitis, in which excessive host immune responses are responsible for the aggravation. Antibacterial activity, along with immunomodulatory effects, has been demonstrated to collectively contribute to the observed efficacy (20, 21). Antibiotics are ineffective against viruses (22, 23), but macrolides have been reported to have immunomodulatory effects against viral infections. Overall, the immunomodulatory effects of macrolides are effective in treating various inflammatory conditions, regardless of the presence or absence of bacterial infections.

This study aimed to select a derivative that exhibited immunomodulatory effects equivalent to or superior to that of EM without antibacterial activity and elucidate the mechanism of this activity. Many studies demonstrated the immunomodulatory effect of EM using changes in cytokine production as an indicator. However, when we examined it from the same perspective, EM982 showed immunomodulatory activity toward all cell lines used in this study, whereas EM decreased cytokine production only in PMA-untreated THP-1 cells. Three previous studies have reported the effect of EM on cytokine production in THP-1 cells: EM inhibited *IL6* transcription in PMA-untreated THP-1 cells by approximately 20% at the mRNA level (24), EM had no significant effect on TNF-α and IL-6 production in PMA-differentiated THP-1 cells at the protein level (25), and EM decreased IL-8 production in THP-1 cells differentiated with 1,25-dihydroxy vitamin D3 by approximately 60% at the protein level (26). These findings suggest that the effect of EM differs depending on the differentiation method even in the same cell type.

Many previous publications examining the immunomodulatory effects of macrolides focused on intracellular signaling pathways for mechanistic analyses. In this study, we found that EM982 inhibited NF-κB activity by inhibiting IKKβ and IκBα phosphorylation, leading to decreased cytokine production, whereas transcription of signaling molecules was not affected. EM982 did not affect the phosphorylation of p38, ERK, or JNK. In this regard, several studies have reported that phosphorylation of these three molecules, especially ERK, was inhibited by macrolides (27, 28). Our findings suggest that EM982 exerts its immunomodulatory action by a somewhat different mechanism or strength than EM. Kanoh *et al.* concluded in their review that the effects of macrolide on signaling pathways are indeed polymodal (12). First, this may be due to the complexity of the signaling pathway. For example, NF-κB is mainly activated by signals from IKKβ, and it also receives signals from mitogen-activated protein kinases, including p38, ERK, and JNK, which function mainly upstream of AP-1 (12, 29). In addition, immune cells collect and integrate information from multiple pathogen-associated molecular patterns and host-derived factors such as cytokines, leading to crosstalk among signaling pathways (30). Second, it may be because the effects of macrolides are "immunomodulatory" rather than simply immunosuppressive. Several reports have shown that the effects of macrolides on signaling pathways have temporal variability; that is, macrolides suppress activation of signaling molecules, followed by a shift to promotion and, finally, return to baseline (31). Finally, as each tissue and cell activates different signaling molecules (32), it is reasonable to expect that the results will vary depending on the timing and target of the analysis.

Macrolides and their derivatives affect various host immune functions as well as cytokine production (9, 12, 27, 33). The immunomodulatory effects on neutrophils include the promotion of apoptosis, inhibition of migration, and suppression of adhesion molecule expression *in vivo* (9). Given that neutrophils are the major infiltrating cells in inflammatory lesions in diffuse pan-bronchiolitis and chronic obstructive pulmonary disease, in which long-term low-dose macrolide administration is effective (8), EM982 may also target neutrophil function. Further studies are needed to elucidate the precise immunomodulatory effects of EM982 and its derivatives.

Although the immunomodulatory effects of EM have been clinically applied in the treatment of non-infectious inflammatory respiratory diseases, the clinical application of EM in this context is associated with the disadvantage of “selective pressure” exerted by the antibacterial action of EM in the human body. Selective pressure eliminates or suppresses susceptible bacteria, creating an environment where drug-resistant bacteria dominate and spread (34). Studies have revealed a positive correlation between antibiotic use and detection rates of drug-resistant bacteria (35, 36, 37, 38). Therefore, reducing the use of “antibiotics” to combat AMR is crucial. Further, utilizing derivatives that retain immunomodulatory activity but lack antibacterial activity by modifying their chemical structures is essential. In this study, the antibacterial activity of EM982 was considerably weaker than that of EM, suggesting the exertion of a weaker selective pressure by EM982. Considering the risk of AMR, further studies are necessary to investigate the non-antibacterial macrolide EM982 as a possible alternative to EM.

## Experimental procedures

Supporting information (SI) includes information on reagents (EM, EM derivatives, and antibodies) and cytokine assay.

### Antibacterial activity assays using EM and EM derivatives

The derivatives were used at a final concentration of 10 μg/ml to screen for EM derivatives without antibacterial activity. For the measurement of the MICs of EM and EM982 against NILS6, 2-fold serial dilutions of the macrolides were prepared. Aliquots (4 μl) of the bacteria culture (optical density [OD_600_] at 600 nm = 0.1) were added into 196 μl of trypticase soy broth (TSB; BD Biosciences) supplemented with DMSO (control), EM, or EM derivatives, and incubated at 37 °C for 24 h. After incubation, OD_600_ was measured using a Multiskan FC Microplate Photometer (Thermo Fisher Scientific), and bacterial growth in the macrolide-treated groups was calculated relative to that in the control group. Other species and strains of bacteria in Table S1, listing combinations of bacteria, media, and culture conditions, were used similarly.

### THP-1 cell culture and stimulation

Monocytic cell line THP-1 was maintained in Roswell Park Memorial Institute (RPMI) 1640 medium (Fujifilm WakoPure Chemical Corporation) supplemented with 10% fetal bovine serum (FBS), 100 U/ml penicillin, and 100 μg/ml streptomycin (Fujifilm Wako Pure Chemical Corporation) at 37 °C in 5% CO_2_. The cells were seeded in 24-well culture plates at a density of 5 × 10^5^ cells/well or in 12-well culture plates at a density of 1 × 10^6^ cells/well in RPMI 1640 medium supplemented with 200 nM PMA (Cayman Chemical) for 48 h to differentiate into macrophage-like cells (THP-1 macrophages). After differentiation, the cells were washed in PBS and cultured in FBS-free RPMI 1640 medium for 24 h. Thereafter, they were treated with 10 μg/ml EM or 1 to 10 μg/ml EM982 for 2 h. Subsequently, the cells were stimulated with five or 100 ng/ml LPS derived from *Escherichia coli* strain 055: B5 (Merck) for the indicated time. Culture and stimulation of PMA-untreated THP-1 cells shown in Fig. S4 are described in SI.

### Real-time RT-PCR

THP-1 macrophages were stimulated with 5 ng/ml LPS for 9 h in the presence or absence of 10 μg/ml EM or EM982. Total RNA was extracted using 1 ml of TRI reagent (Molecular Research Center) according to the manufacturer’s protocol. Complementary DNA was synthesized using the ReverTra Ace qPCR RT Master Mix with gDNA remover (TOYOBO), according to the manufacturer’s protocol. Real-time PCR was performed on a StepOnePlus real-time PCR system (Thermo Fisher Scientific) using TaqMan Fast Advanced Master Mix (Thermo Fisher Scientific) and TaqMan Array Plate (#4418838; Thermo Fisher Scientific), according to the manufacturer’s instructions. The plate was pre-loaded with primers and TaqMan probes for 12 reference genes and 84 TLR signaling genes (Table S2). *HPRT1* was used as the reference gene for the normalization of mRNA levels, because of its low intergroup variation.

### SEAP reporter assay

HEK-Blue cells expressing human Toll-like receptor 4 (HEK-Blue hTLR4) were obtained from InvivoGen (San Diego, CA, USA). After binding of TLR4 ligand, such as LPS, the cells secrete secretory embryonic alkaline phosphatase (SEAP) following activation of transcription factors NF-κB and AP-1. The cells were maintained in Dulbecco’s modified Eagle’s medium (Fujifilm Wako Pure Chemical Corporation) supplemented with 10% FBS, 100 U/ml penicillin, 100 μg/ml streptomycin, 100 μg/ml normocin (InvivoGen), and HEK-Blue Selection (InvivoGen) at 37 °C in 5% CO_2_. The cells were suspended in HEK-Blue Detection (InvivoGen) medium, which contains a SEAP substrate and allows for monitoring of changes in SEAP level, and seeded at a density of 2.5 × 10^4^ cells/180 μl. Then, 20 μl of HEK-Blue Detection supplemented with EM or EM982 (final concentration of 1–10 μg/ml) was added to the wells and incubated for 2 h. After treatment, LPS was added at a final concentration of 5 ng/ml and incubated for 15 h at 37 °C in 5% CO_2_. SEAP activity was measured using a Multiskan FC microplate photometer at 620 nm (*A*_620_). Protocols for THP-1-Blue NF-κB cells are described in SI.

### Western blotting

THP-1 macrophages were stimulated with LPS for 10 to 60 min in the presence or absence of 10 μg/ml EM or EM982. Total protein was extracted using the M-PER Mammalian Protein Extraction Reagent (Thermo Fisher Scientific) supplemented with 1% Halt Protease and Phosphatase Inhibitor Cocktail (Thermo Fisher Scientific). The extracted proteins were suspended in a sample buffer followed by incubation at 95 °C for 5 min. Protein samples were separated using SDS-PAGE on a 10% polyacrylamide gel (Bio-Rad) and transferred onto a polyvinylidene difluoride membrane (Merck). The membrane was blocked with 5% BSA or 5% skim milk (BD Biosciences) in Tris-buffered saline containing Tween 20 (Takara Bio). After blocking, the membrane was incubated with each primary antibody overnight at 4 °C, followed by incubation with horseradish peroxidase-conjugated secondary antibodies for 1 h at room temperature. HRP substrate (Cytiva) was then added, and chemiluminescence was detected using an ImageQuant LAS-4000 mini (Cytiva). The intensities of Western blot signals were quantified using Image Studio Lite (Ver5.2; LI-COR Biosciences) and normalized to those of GAPDH. The graphs show the relative values of phospho-IKKβ and phospho-IκBα relative to those in the LPS-only group following 10 min incubation. Statistical analysis was performed using one-way analysis of variance among the four groups with the same incubation time.

### Statistical analysis

Data were analyzed by one-way analysis of variance with Dunnett’s multiple comparison test using GraphPad Prism version 9.5.1 (GraphPad Software). Statistical significance was set at *p* < 0.05. All data are presented as mean ± standard deviation.

## Data availability

All data are available in the main text or in the supplementary materials.

## Supporting information

This article contains supporting information (39).

## Conflict of interest

The authors declare that they have no known competing financial interests or personal relationships that could have appeared to influence the work reported in this paper.
