# Supplementary material for: A novel 12-membered ring non-antibiotic macrolide EM982 attenuates cytokine production by inhibiting IKKβ and IκBα phosphorylation
Source: J Biol Chem. 2024 May 16;300(6):107384. doi: 10.1016/j.jbc.2024.107384 (PMC11190463; doi:10.1016/j.jbc.2024.107384)
Supplement: Supporting Information [file mmc1.docx]

**Supporting Information

A novel 12-membered ring non-antibiotic macrolide EM982 attenuates cytokine production by inhibiting IKKβ and IκBα phosphorylation**

Rui Saito ^a, b^, Hisanori Domon ^a, c^, Takumi Hiyoshi ^a, c, d^, Satoru Hirayama ^a^, Tomoki Maekawa ^a, c, d^,
Shoji Takenaka ^b^, Yuichiro Noiri ^b^, Akari Ikeda ^e^, Tomoyasu Hirose ^e^, Toshiaki Sunazuka ^e^, and Yutaka Terao ^a, c, *^

^a^ Division of Microbiology and Infectious Diseases, Niigata University Graduate School of Medical and Dental Sciences, Niigata, Japan

^b^ Division of Cariology, Operative Dentistry and Endodontics, Department of Oral Health Science, Niigata University Graduate School of Medical and Dental Sciences, Niigata, Japan

^c^ Center for Advanced Oral Science, Niigata University Graduate School of Medical and Dental Sciences, Niigata, Japan

^d^ Division of Periodontology, Niigata University Graduate School of Medical and Dental Sciences, Niigata, Japan

^e^ Kitasato Institute for Life Sciences, Kitasato University Graduate School of Infection Control Sciences, Kitasato University, Tokyo, Japan

***Corresponding author**: Yutaka Terao

**Email:** [terao@dent.niigata-u.ac.jp](mailto:terao@dent.niigata-u.ac.jp)

This Supporting Information includes:

Figure S1–4, Table S1–2 and supplemental experimental procedures

**Table S1. Bacteria, media, culture conditions, and minimum inhibitory concentrations of EM and EM982 against each bacterium**

| Species and strains | Medium | Culture  Conditions | MICs*^1^ (µg/mL) | |
| --- | --- | --- | --- | --- |
|  |  |  | EM*^2^ | EM982 |
| *Staphylococcus aureus* NILS7 | TSB*^3^ | aerobic | 0.25 | > 64 |
| *S. aureus* NILS8 | TSB | aerobic | 0.25 | > 64 |
| *S. aureus* NILS9 | TSB | aerobic | 1 | > 64 |
| *S. aureus* NILS10 | TSB | aerobic | 0.25 | > 64 |
| *Streptococcus pneumoniae* D39 | TSB | aerobic | 0.0625 | 32 |
| *Streptococcus mutans* MT8148 | BHI*^4^ | aerobic | 0.0625 | > 64 |
| *Porphylomonas gingivalis* ATCC 33277 | Modified GAM*^5^ broth | anaerobic | 0.125 | 32 |
| *Aggregatibacter actinomycetemcomitans*  HK1651 | TSB with 0.6% yeast extract and 0.04% sodium bicarbonate | anaerobic | 0.125 | > 64 |

*1 MICs; minimum inhibitory concentrations

*2 EM; erythromycin

*3 TSB; trypticase soy broth

*4 BHI; brain heart infusion broth. This was obtained from BD Biosciences

*5 GAM; Gifu anaerobic broth, modified. This was obtained from Shimadzu Diagnostics Corporation


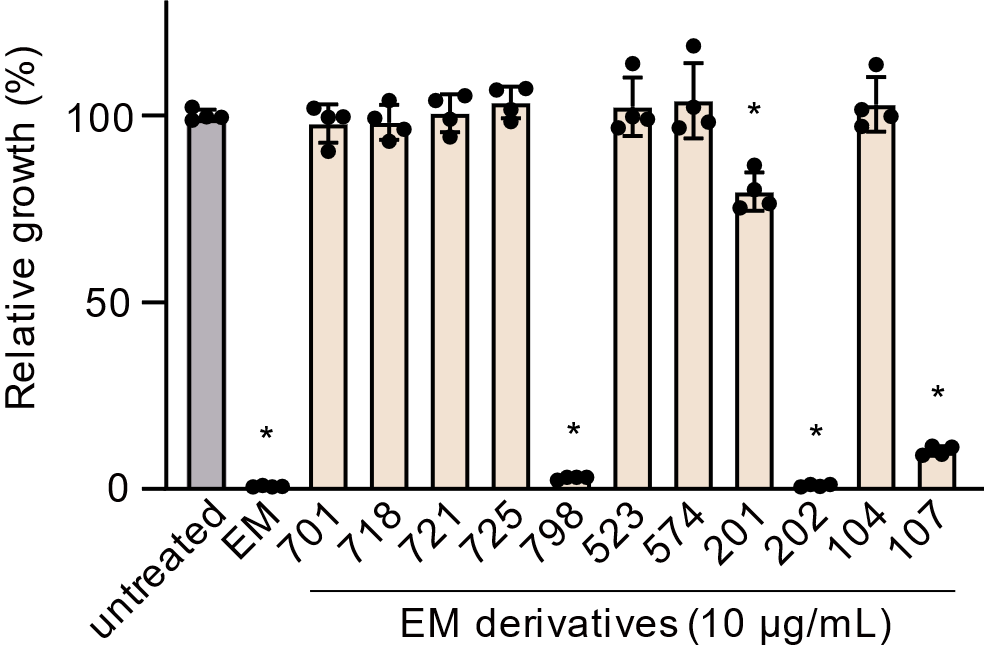
**Figure S1. Effects of other EM derivatives on the proliferation of *S. aureus***The Antibacterial activity of EM derivatives was analyzed. Macrolide-sensitive *S. aureus* strain NILS6 was cultured in the presence or absence of 10 μg/mL EM or EM derivatives for 24 h. Bacterial proliferation was quantified by spectrometry at 620 nm. The data represent the mean ± SD of samples per group and were evaluated by one-way ANOVA with Dunnett's multiple comparisons test. The asterisks indicate significant differences as compared with untreated group

**
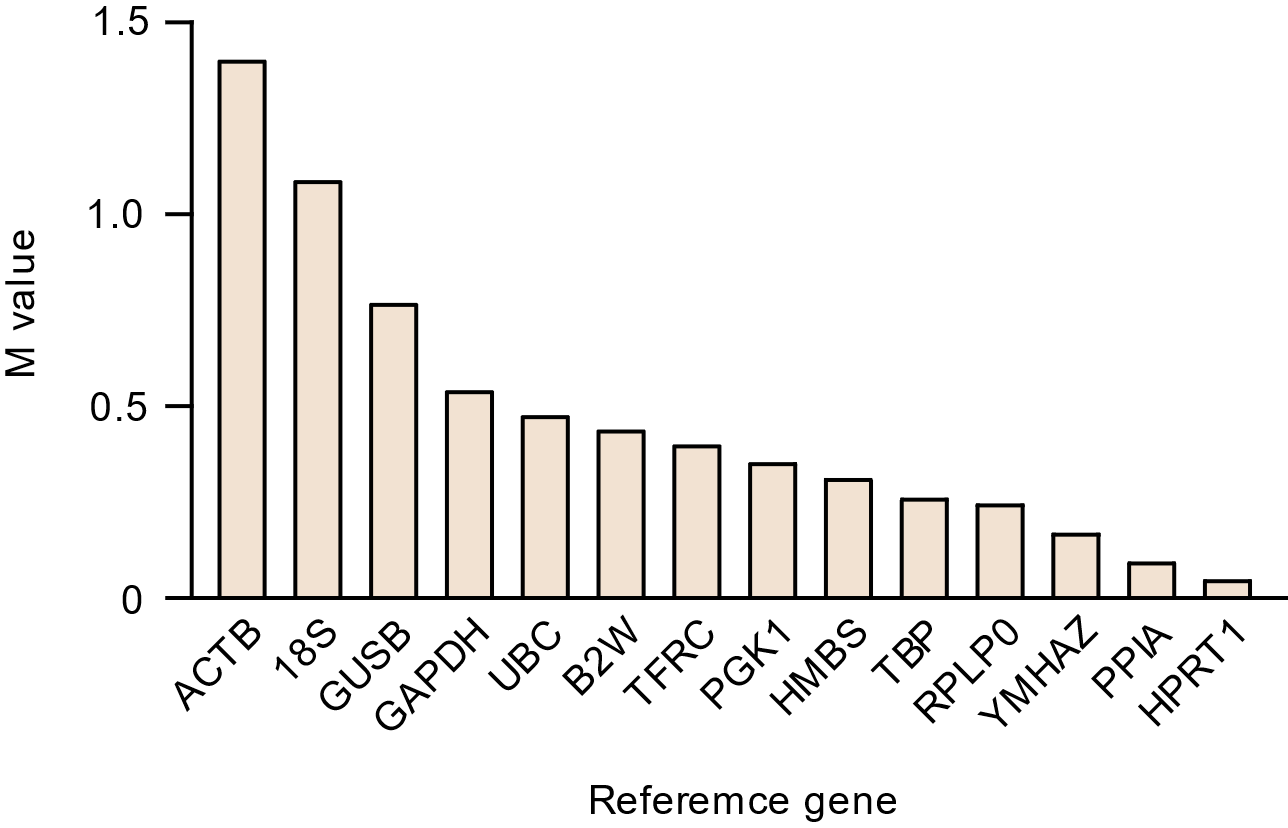
Figure S2. Determination of optimal reference gene**Using the geNorm algorithm approach in the EZR statistical analysis software (39), *HPRT-1* was determined to be the most stable among the 12 reference genes.

**Figure S3. EM982 downregulated NF-κB pathway in THP-1-Blue cells**

**
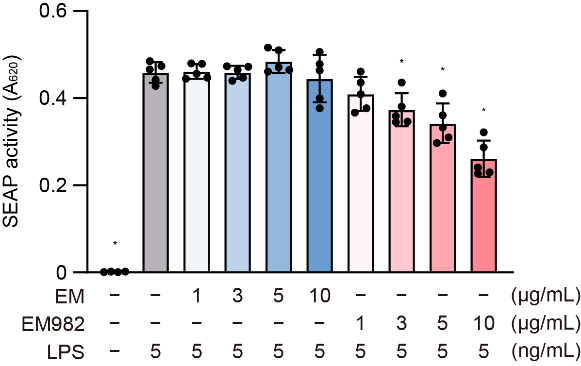
**THP-1-Blue cells were cultured with 1–10 μg/mL EM or EM982 for 2 h prior to stimulation with 5 ng/mL lipopolysaccharide (LPS) for 15 h. The culture supernatant was added to QUANTI-Blue and incubated for 1 h. The activity of secretary embryonic alkaline phosphatase (SEAP) was quantified by spectrometry at 620 nm. The data represent the mean ± SD of samples per group and were evaluated by one-way ANOVA with Dunnett's multiple comparisons test. The asterisks indicate significant differences as compared with the LPS-only group (**P* < 0.05)

**Figure S4. EM and EM982 inhibited IL-6 and IL-8 productions by** **phorbol myristate acetate (PMA)-untreated THP-1 cells**


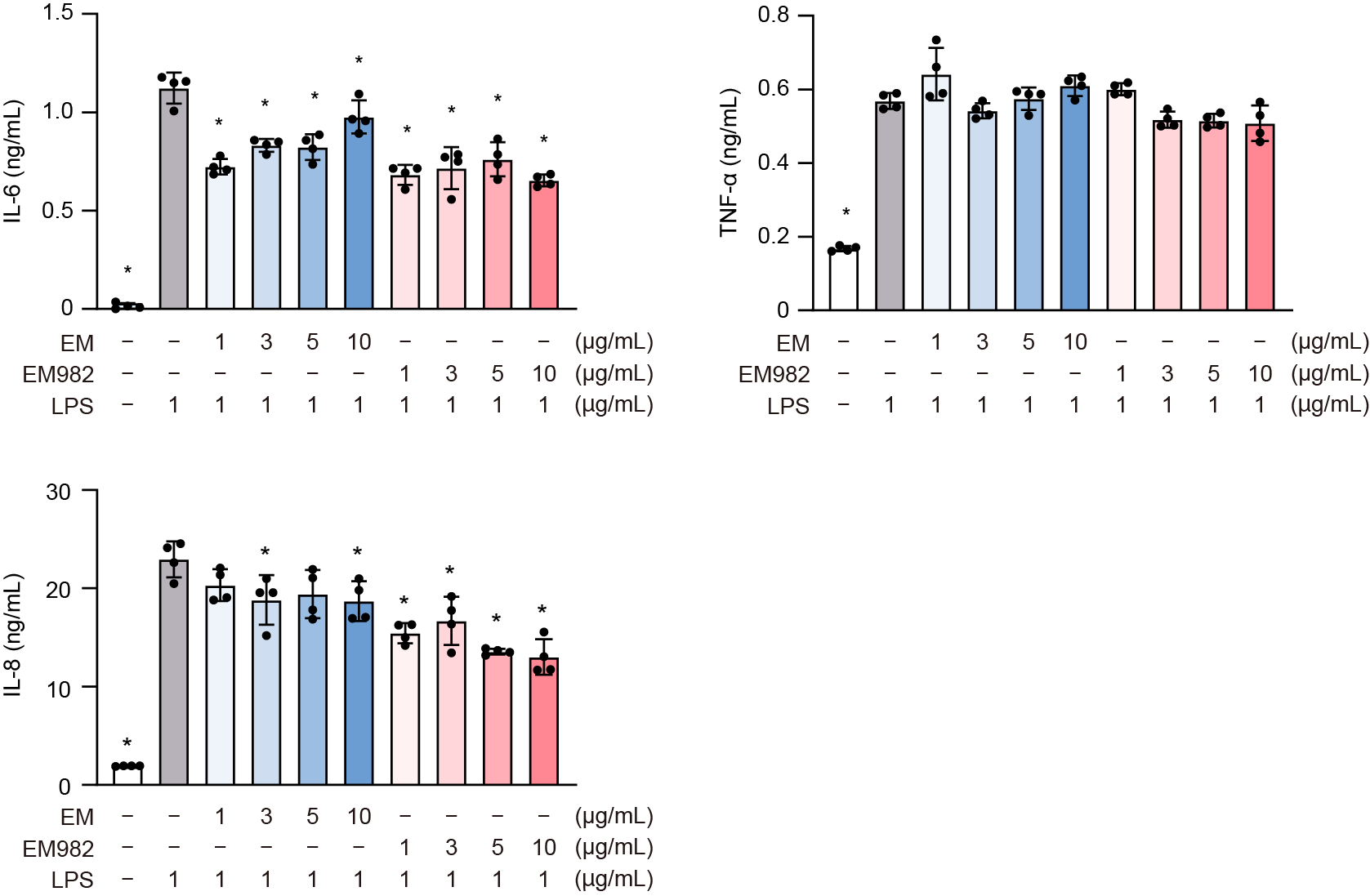
PMA-untreated THP-1 cells were incubated in the presence or absence of 1–10 µg/mL EM or EM982 for 2 h prior to stimulation by 1 µg/mL LPS for 12 h. Cytokine levels in the culture supernatant were measured by ELISA. The data represent the mean ± SD of samples per group and were evaluated by one-way ANOVA with Dunnett's multiple comparisons test. The asterisks indicate significant differences as compared with the LPS-only group (**P* < 0.05)

**Supplemental Experimental Procedures**

**Erythromycin (EM) and EM derivatives**

Erythromycin was purchased from Fujifilm Wako Pure Chemical Corporation. The synthesis methods for the EM derivatives are provided in the next section. Erythromycin and derivatives were dissolved in DMSO (Nacalai Tesque) and stored at -20 °C.

**Synthesis of EM derivatives**

Unless otherwise noted, reagents and solvents were commercially available and used without further purification. All dry solvents such as MeOH and THF were purchased from Kanto Chemical Co., Inc. For thin layer chromatography (TLC) analysis, precoated silica gel plates with a fluorescent indicator (Merck 60 F254) were used. Flash chromatography was carried out with Kanto Chemical silica gel (Kanto Chemical, silica gel 60N, spherical neutral, 0.040-0.050 mm) or Merck silica gel 230-400 mesh ASTM (60N, 0.040-0.063 mm). Optical rotations were measured on a JASCO P-1010 polarimeter. Melting point (m.p.) was measured on an SRS MPA-100 optimelt. Infrared (IR) spectra were recorded on a Horiba FT-210 spectrometer. NMR spectra were measured on a JEOL JNM-ECA-500 spectrometer with ^1^H NMR at 500 MHz and ^13^C NMR at 125 MHz. Chemical shifts were reported in ppm from the internal solvent peaks for chloroform-*d_1_* (CDCl_3_) (^1^H; δ= 7.26 ppm, ^13^C; δ= 77.16 ppm. ^1^H NMR data were reported as follows: chemical shift (integration, multiplicity (s = singlet, d = doublet, t = triplet, m = multiplet, br = broad), coupling constants (Hz)). The high-resolution mass spectra (HRMS) were performed on a JEOL JMS-700 MStation and JEOL JMS-T100LP.

(2*S*,3*R*,4*S*,6*R*)-4-(Dimethylamino)-2-(((1*S*,2*S*,3*R*,6*R*,7*S*,9*R*,12*R*)-3-((4*R*,5*R*)-5-ethyl-2-(4-methoxyphenyl)-4-methyl-1,3-dioxolan-4-yl)-7-(((2*R*,4*R*,5*S*,6*S*)-5-hydroxy-4-methoxy-4,6-dimethyltetrahydro-2H-pyran-2-yl)oxy)-2,6,8,10,12-pentamethyl-5-oxo-4,13-dioxabicyclo[8.2.1]tridecan-9-yl)oxy)-6-methyltetrahydro-2H-pyran-3-yl acetate (**1**).

To a solution of EM900 (500 mg, 0.70 mmol) in acetone (7 mL, 0.1 M) was added acetic anhydride (328 µL, 3.48 mmol, 5 equiv.) at room temperature under N_2_ atmosphere. After being stirred for 2 h, the reaction mixture was quenched with sat. NaHCO_3_ aq. (15 mL), and extracted with ethyl acetate (15 mL x3). The combined organic layer was dried over Na_2_SO_4_, filtered, and concentrated *in vacuo* to obtain a crude product. To a solution of the crude (0.70 mmol) in dichloromethane (7 mL, 0.1 M) was added *p*-anisaldehyde dimethyl acetal (544 µL, 3.2 mmol, 4.5 equiv.) and pyridinium *p*-toluenesulfonate (1.98 g, 7.9 mmol, 11.3 equiv.) at room temperature under N_2_ atmosphere. After being stirred for 22.5 h, the reaction mixture was quenched with sat. NaHCO_3_ aq. (15 mL), and extracted with ethyl acetate (15 mL x3). The combined organic layer was dried over Na_2_SO_4_, filtered, and concentrated *in vacuo*. The crude was purified with column chromatography (chloroform/methanol/NH_3_ aq. = 20/1/0.1) to obtain a compound **1** (456 mg, 67% yield) as a colorless solid. IR(diamond prism)νcm^-1^: 2973, 2936, 2361, 2341, 1739, 1709, 1615, 1518, 1457, 1374, 1246, 1167, 1114, 1056, 1015, 1003, 937, 871, 832, 755, 664, 611, 594, 542, 514, 496, 485, 472, 459, 445, 435, 422. [α]^24^_D_: -402.68 (c 1.0, CHCl_3_). ^1^H NMR (500 MHz, CDCl_3_) δ(ppm): 7.36 (d, *J* = 8.0 Hz, 2H), 6.92 (d, *J* = 6.9 Hz, 2H), 6.88 (d, *J* = 8.6 Hz, 2H), 5.77 (s, 1H), 4.79-4.75 (m, 2 H), 4.67 (d, *J* = 4.0 Hz, 1H), 4.32 (d, *J* = 6.9 Hz, 1H), 4.11 (q, *J* = 7.5 Hz, 1H), 4.01 (br s, 1H), 3.81-3.79 (m, 1H), 3.79 (s, 3H), 3.63 (dd, *J* = 9.2, 4.6 Hz, 1H), 3.48-3.44 (m, 2H), 3.27 (s, 1H), 3.23 (s, 3H), 2.99 (t, *J* = 9.2 Hz, 1H), 2.71 (td, *J* = 11.5, 4.0 Hz, 1H), 2.36-2.33 (m, 2H), 2.28-2.25 (m, 10H), 2.18-2.17 (m, 1H), 2.07 (s, 3H), 2.04 (s, 1H), 1.43-1.33 (m, 7H), 1.71 (dd, *J* = 13.2, 2.3 Hz, 1H), 1.31 (d, *J* = 6.3 Hz, 3H), 1.25 (t, *J* = 6.3 Hz, 3H), 1.22-1.20 (m, 9H), 1.17 (dd, *J* = 6.3, 3.4 Hz, 1H), 1.03 (t, *J* = 7.5 Hz, 3H), 0.98 (d, *J* = 7.5 Hz, 1H), 0.95 (d, *J* = 6.9 Hz, 3H). ^13^C NMR (125 MHz, CDCl_3_) δ(ppm): 174.0, 169.9, 160.2, 150.0, 136.1, 133.8, 129.6, 128.7, 123.9, 113.9, 113.5, 101.5, 97.8, 83.5, 83.4, 80.7, 78.5, 78.4, 72.6, 71.8, 68.9, 65.5, 63.2, 55.4, 53.9, 49.4, 47.1, 40.8, 35.4, 35.0, 31.9, 30.9, 29.5, 29.4, 24.7, 21.9, 21.7, 21.6, 21.1, 18.1, 14.3, 12.2, 11.8, 9.9. *m/z*: 878.5273 [M+H]^+^, calcd for C_47_H_76_NO_14_: 878.5266 [M+H]^+^.

(2*S*,3*R*,4*S*,6*R*)-2-(((1*S*,2*S*,3*R*,6*R*,7*S*,9*R*,12*R*)-7-(((2*R*,4*R*,5*S*,6*S*)-5-(((*N*-(3-(((benzyloxy)carbonyl)amino)propyl)sulfamoyl)carbamoyl)oxy)-4-methoxy-4,6-dimethyltetrahydro-2H-pyran-2-yl)oxy)-3-((4*R*,5*R*)-5-ethyl-2-(4-methoxyphenyl)-4-methyl-1,3-dioxolan-4-yl)-2,6,8,10,12-pentamethyl-5-oxo-4,13-dioxabicyclo[8.2.1]tridecan-9-yl)oxy)-4-(dimethylamino)-6-methyltetrahydro-2H-pyran-3-yl acetate (**2**).

To a solution of Cbz-amine (213 mg, 0.70 mmol, 2.9 equiv.) in dichloromethane (5.4 mL) was added diisopropylethylamine (120.0 µL, 0.70 mmol, 2.9 equiv.) at 0 ^o^C under N_2_ atmosphere, and stirred for 30 min as a premix. To another solution of the compound **1** (208 mg, 0.24 mmol) in dichloromethane (4.7 mL, 0.05 M) was added chlorosulfonylisocyanate (30.9 µL, 0.36 mmol, 1.5 equiv.) at 0 ^o^C under N_2_ atmosphere. After being stirred for 10 min, the reaction mixture was added dropwise with the premix amine at 0 ^o^C. After being stirred for 1.5 h, the reaction mixture was quenched with sat. NaHCO_3_ aq. (15.0 mL), and extracted with CHCl_3_ (10 mL x3). The combined organic layer was dried over Na_2_SO_4_, filtered, and concentrated *in vacuo*. The crude was purified with column chromatography (chloroform/methanol/NH_3_ aq. = 5/1/0.1) to obtain a compound **2** (155 mg, 55% yield) as a colorless solid. IR(diamond prism)νcm^-1^: 3335, 2976, 2937, 1707, 1658, 1615, 1518, 1455, 1376, 1336, 1249, 1214, 1167, 1119, 1070, 1016, 890, 825, 748, 665, 645, 594, 580, 554, 528, 509, 491, 473, 458, 443, 428, 415, 405. [α]^23^_D_: -371.60 (c 1.0, CHCl_3_). ^1^H NMR (500 MHz, CDCl_3_) δ(ppm): 7.62 (br s, 1H), 7.38-7.28 (m, 8H), 6.92 (d, *J* = 8.0 Hz, 2H), 6.88 (d, *J* = 8.6 Hz, 2H), 5.77 (s, 1H), 5.40 (br s, 1H), 5.06 (s, 2H), 4.79 (d, *J* = 6.9 Hz, 1H), 4.67 (d, *J* = 4.0 Hz, 1H), 4.49 (d, *J* = 9.7 Hz, 1H), 4.31-4.25 (m, 2H), 4.12 (d, *J* = 9.7 Hz, 1H), 3.80 (s, 1H), 3.79 (s, 3H), 3.72 (dq, *J* = 6.9, 6.9 Hz, 2H), 3.63 (dd, *J* = 8.6, 4.6 Hz, 1H), 3.51-3.50 (m, 1H), 3.36-3.28 (m, 2H), 3.20 (s, 2H), 3.16 (s, 3H), 3.00 (br s, 2H), 2.31 (m, 7H), 2.24 (d, *J* = 14.9 Hz, 1H), 2.07 (s, 3H), 1.78-1.56 (m, 5H), 1.43 (m, 2H), 1.37 (m, 13H), 1.32 (t, *J* = 7.5 Hz, 3H), 1.22 (d, *J* = 6.3 Hz, 3H), 1.18 (d, *J* = 4.8 Hz, 3H), 1.10-1.08 (m, 5H), 1.03 (t, *J* = 7.5 Hz, 3H), 0.92 (d, *J* = 6.9 Hz, 3H). ^13^C NMR (125 MHz, CDCl_3_) δ(ppm): 173.9, 173.5, 170.0, 160.3, 156.7, 138.0, 136.8, 133.7, 128.6, 128.22, 128.15, 127.9, 113.9, 113.5, 101.5, 98.0, 85.0, 83.3, 81.2, 78.9, 73.4, 71.4, 68.7, 66.7, 66.6, 63.4, 63.3, 63.1, 55.42, 55.38, 52.7, 49.3, 47.0, 41.1, 40.8, 40.4, 38.2, 35.6, 35.0, 30.4, 29.7, 24.6, 21.9, 21.6, 21.3, 21.0, 18.83, 18.75, 17.4, 16.3, 12.2, 11.8, 11.6, 10.0. *m/z*: 1191.6019 [M+H]^+^, calcd for C_59_H_91_N_4_O_19_S: 1191.5998 [M+H]^+^.

(2*S*,3*S*,4*R*,6*R*)-6-(((1*S*,2*S*,3*R*,6*R*,7*S*,9*R*,12*R*)-9-(((2*S*,3*R*,4*S*,6*R*)-4-(dimethylamino)-3-hydroxy-6-methyltetrahydro-2H-pyran-2-yl)oxy)-3-((4*R*,5*R*)-5-ethyl-2-(4-methoxyphenyl)-4-methyl-1,3-dioxolan-4-yl)-2,6,8,10,12-pentamethyl-5-oxo-4,13-dioxabicyclo[8.2.1]tridecan-7-yl)oxy)-4-methoxy-2,4-dimethyltetrahydro-2H-pyran-3-yl (*N*-(3-(((benzyloxy)carbonyl)amino)propyl)sulfamoyl)carbamate (**3**).

To a solution of the compound **2** (177 mg, 0.15 mmol) in methanol (1.5 mL, 0.1 M) was heated at 50 ^o^C for 22 h, then cooled to room temperature. The reaction mixture was concentrated *in vacuo*. The crude was purified with column chromatography (chloroform/methanol/NH_3_ aq. = 5/1/0.1) to obtain a compound **3** (150 mg, 88% yield) as a colorless solid. The obtained novel 12-membered ring non-antimicrobial macrolide **3** was named as an **EM982**. IR(diamond prism)νcm^-1^: 3330. 2973, 2936, 2879, 2364, 1714, 1658, 1616, 1517, 1457, 1376, 1339, 1250, 1168, 1072, 1015, 913, 828, 743, 698, 647, 592, 519, 506, 494, 473, 457, 446, 424. [α]^23^_D_: -480.32 (c 1.0, CHCl_3_). ^1^H NMR (500 MHz, CDCl_3_) δ(ppm): 7.59 (br s, 1H), 7.39-7.28 (m, 8H), 6.91 (d, *J* = 8.0 Hz, 2H), 6.88 (d, *J* = 8.6 Hz, 2H), 5.78 (s, 1H), 5.40 (br s, 1H), 5.07 (s, 2H), 4.696-4.692 (m, 1H), 4.45 (d, *J* = 9.7 Hz, 1H), 4.32-4.29 (m, 2H), 4.08 (d, *J* = 9.7 Hz, 1H), 3.799 (s, 2H), 3.797 (s, 3H), 3.64 (dd, *J* = 9.2, 4.0 Hz, 1H), 358 (br s, 1H), 9.74 (d, *J* = 9.7 Hz, 1H), 3.29-3.26 (m, 4H), 3.18 (s, 1H), 3.13 (s, 2H), 2.99 (m, 2H), 2.72 (m, 6H), 2.39-2.25 (m, 4H), 2.01-1.98 (m, 1H), 1.72-1.71 (m, 4H), 1.43-1.38 (m, 7H), 1.25-1.21 (m, 9H), 1.18 (d, *J* = 5.7 Hz, 3H), 1.11-1.10 (m, 5H), 1.08-1.06 (m, 1H), 1.05-1.02 (m, 10H). ^13^C NMR (125 MHz, CDCl_3_) δ(ppm): 173.8, 173.5, 160.2, 156.6, 136.9, 133.7, 129.6, 128.6, 128.3, 128.1, 127.9, 113.8, 113.5, 101.5, 97.9 91.5, 84.8, 83.6, 83.4, 81.1, 79.2, 73.2, 71.3, 68.9, 68.2, 66.6, 64.5, 63.3, 55.4, 49.3, 49.2, 46.9, 41.3, 40.3, 39.9, 38.5, 35.4, 35.0, 29.8, 29.6, 24.7, 21.9, 21.6, 21.5, 21.2, 20.8, 17.6, 16.7, 13.6, 12.3, 11.8, 10.5. *m/z*:1149.5897 [M+H]^+^, calcd for C_57_H_89_N_4_O_18_S: 1149.5893 [M+H]^+^

**Bacterial strain and culture**

The EM-sensitive *Staphylococcus aureus* strain NILS6 used in this study was a clinical isolate obtained from a patient with staphylococcal pneumonia. NILS6 was grown in TSB (BD Biosciences) for 12–16 h at 37 °C under aerobic conditions. The overnight culture was seeded at a dilution of 1:100 in fresh TSB medium and grown until the exponential growth phase (optical density at 600 nm = 0.1) was reached. The cultures were then used for antibacterial assays.

**PMA-untreated THP-1 cell culture**

Monocytic cell line THP-1 was maintained in RPMI 1640 medium supplemented with 10% fetal bovine serum, 100 U/mL penicillin, and 100 µg/mL streptomycin at 37 °C in 5% CO_2_. The cells were seeded in 24-well culture plates at a density of 5 × 10^5^ cells/well in RPMI 1640 medium supplemented without PMA.

**Cytokine assay**

To screen for EM derivatives with immunomodulatory effects, we stimulated THP-1 macrophages with 100 ng/mL LPS for 8 h in the presence or absence of 10 µg/mL EM and EM derivatives. To assess the effect of EM982 on cytokine production, we stimulated THP-1 macrophages and PMA-untreated THP-1 cells with 5 ng/mL LPS for 12 h in the presence or absence of 1–10 µg/mL EM or 1–10 µg/mL EM982. TNF-α, IL-6, IL-8, and IL-10 levels in the culture supernatant were measured using ELISA kits (BioLegend) following the manufacturer’s protocol.

**SEAP reporter assay with THP-1-Blue cells**

THP-1-Blue NF-κB cells were obtained from InvivoGen. After binding of TLR4 ligands, such as LPS, the cells secrete secretory embryonic alkaline phosphatase (SEAP) following activation of the transcription factors NF-κB. The cells were maintained in Roswell Park Memorial Institute (RPMI) 1640 medium (Fujifilm Wako) supplemented with 10% fetal bovine serum, 100 U/mL penicillin, 100 µg/mL streptomycin (Fujifilm Wako), 100 µg/mL normocin (InvivoGen), and 10 µg/mL blastcidin (InvivoGen) at 37 °C in 5% CO_2_. The cells were seeded in a 96-well culture plate at a density of 1 × 10^5^ cells/180 µL. Then, 20 µL of medium supplemented with EM or EM982 (final concentration of 1–10 µg/mL) was added to the wells and incubated for 2 h. After treatment, LPS derived from *Escherichia coli* strain 055: B5 (Merck) was added at a final 5 ng/mL concentration and incubated for 15 h at 37 °C in 5% CO_2_. Culture supernatant (10 µg/mL) was added to 90 µL of QUANTI-Blue (InvivoGen), which contains SEAP substrate and results in blue coloration to SEAP activity, and incubated at 37 °C for 1 h. SEAP activity was measured using Multiskan FC Microplate Photometer (Thermo Fisher Scientific) at 620 nm (*A*_620_).

**Antibodies for Western blotting**

The primary antibodies used in this study were as follows: anti-phospho-IKKα/IKKβ mAb (#2078) and anti-phospho-IκBα mAb (#9246) from Cell Signaling Technology, anti-phospho-p38 polyclonal Ab (#28796-1-AP), anti-phospho-ERK1/2 polyclonal Ab (#28733-1-AP), and anti-phospho-JNK recombinant Ab (#80024-1-RR) from Proteintech, anti-GAPDH mAb (#ab8245) from Abcam. These were used at a dilution of 1:1000–1:5000. The secondary antibodies used in this study were anti-mouse IgG, HRP-conjugated Ab (#7076) and anti-rabbit IgG, HRP-conjugated Ab (#7074) from Cell Signaling Technology. These were used at a dilution of 1:3000.

**Table S2. Primers and probes of TaqMan Array**

| Target gene | Assay ID | Target gene | Assay D |
| --- | --- | --- | --- |
| *18S* | Hs99999901_s1 | *MAPK14* | Hs00234085_m1 |
| *GAPDH* | Hs99999905_m1 | *MAPK8* | Hs01548508_m1 |
| *HPRT1* | Hs99999909_m1 | *MAPK9* | Hs00177102_m1 |
| *GUSB* | Hs99999908_m1 | *MYD88* | Hs00182082_m1 |
| *ACTB* | Hs99999903_m1 | *NFKB1* | Hs00765730_m1 |
| *B2W* | Hs99999907_m1 | *NFKB2* | Hs00174517_m1 |
| *RPLP0* | Hs99999902_m1 | *NFKBIA* | Hs00153283_m1 |
| *HMBS* | Hs00609297_m1 | *NFKBIB* | Hs00182115_m1 |
| *TBP* | Hs99999910_m1 | *NFKBIE* | Hs00234431_m1 |
| *PGK1* | Hs99999906_m1 | *PIK3C2A* | Hs00153223_m1 |
| *UBC* | Hs00824723_m1 | *PIK3C2B* | Hs00153248_m1 |
| *PPIA* | Hs99999904_m1 | *PIK3C2G* | Hs00362135_m1 |
| *TFRC* | Hs99999911_m1 | *PIK3C3* | Hs00176908_m1 |
| *YWHAZ* | Hs00237047_m1 | *PIK3CA* | Hs00180679_m1 |
| *ATF2* | Hs00153179_m1 | *PIK3CB* | Hs00927728_m1 |
| *ATF4* | Hs00909568_g1 | *PIK3CD* | Hs00192399_m1 |
| *BTK* | Hs00163761_m1 | *PIK3R1* | Hs00381459_m1 |
| *CD14* | Hs02621496_s1 | *PIK3R2* | Hs00178181_m1 |
| *CHUK* | Hs00175141_m1 | *PIK3R3* | Hs00177524_m1 |
| *CREB1* | Hs00231713_m1 | *PIK3R4* | Hs00300795_m1 |
| *CREB3* | Hs00197255_m1 | *PIK3R5* | Hs00204803_m1 |
| *CREB3L4* | Hs00370116_m1 | *RAC1* | Hs01025984_m1 |
| *ECSIT* | Hs00213167_m1 | *REL* | Hs00968436_m1 |
| *IKBKB* | Hs00233287_m1 | *RELA* | Hs00153294_m1 |
| *IKBKE* | Hs01063858_m1 | *RELB* | Hs00232399_m1 |
| *IKBKG* | Hs00415849_m1 | *RIPK1* | Hs00169407_m1 |
| *IL1RL1* | Hs00249384_m1 | *RIPK2* | Hs01572690_m1 |
| *IRAK1* | Hs00155570_m1 | *RIPK3* | Hs00179132_m1 |
| *IRAK2* | Hs00176394_m1 | *RNF216* | Hs00219248_m1 |
| *IRAK3* | Hs00200502_m1 | *SIGIRR* | Hs00222347_m1 |
| *IRAK4* | Hs00211610_m1 | *TANK* | Hs00370305_m1 |
| *IRF3* | Hs01547282_m1 | *TBK1* | Hs00179410_m1 |
| *IRF7* | Hs00185375_m1 | *TICAM1* | Hs01090712_m1 |
| *IRF8* | Hs01128710_m1 | *TICAM2* | Hs00899439_m1 |
| *JUN* | Hs99999141_s1 | *TIRAP* | Hs00364644_m1 |
| *LBP* | Hs00188074_m1 | *TLR1* | Hs00413978_m1 |
| *LY96* | Hs00209771_m1 | *TLR10* | Hs01675179_m1 |

| *MAP2K3* | Hs00177127_m1 | *TLR2* | Hs01014511_m1 |
| --- | --- | --- | --- |
| *MAP2K6* | Hs00992389_m1 | *TLR3* | Hs00152933_m1 |
| *MAP2K7* | Hs00178198_m1 | *TLR4* | Hs00152939_m1 |
| *MAP3K3* | Hs00176747_m1 | *TLR5* | Hs00152825_m1 |
| *MAP3K7* | Hs00177373_m1 | *TLR6* | Hs00271977_s1 |
| *MAP3K7IP1* | Hs00196143_m1 | *TLR7* | Hs00152971_m1 |
| *MAP3K7IP2* | Hs00248373_m1 | *TLR8* | Hs00152972_m1 |
| *MAPK10* | Hs00373461_m1 | *TLR9* | Hs00152973_m |
| *MAPL11* | Hs00177101_m1 | *TOLLIP* | Hs01553188_m1 |
| *MAPK12* | Hs00268060_m1 | *TRAF6* | Hs00377558_m1 |
| *MAPK13* | Hs00234085_m1 | *UBE2N* | Hs00854751_s1 |
